# The Role of Environmental Enrichment and Back Fat Depth in the Intensity of Aggressive Behavior Performed by Sows during the Establishment of the Dominance Hierarchy

**DOI:** 10.3390/ani13050825

**Published:** 2023-02-24

**Authors:** Maria Costanza Galli, Martyna E. Lagoda, Flaviana Gottardo, Barbara Contiero, Laura A. Boyle

**Affiliations:** 1 Department of Animal Medicine, Production and Health, University of Padova, Viale dell’Università 16, Agripolis, 35020 Legnaro, Italy; 2 Pig Development Department, Teagasc Animal and Grassland Research and Innovation Centre, Moorepark, Fermoy, P61 C997 Cork, Ireland

**Keywords:** gestating sows, environmental enrichment, group-housing, welfare, aggressive behavior

## Abstract

**Simple Summary:**

The need to establish a social hierarchy represents a period of severe stress for sows when they are introduced into new groups because of the associated aggressive behavior. The aim of this study was to determine the effect of providing access to straw in racks and manila ropes (IMPROVED) on aggressive behavior after mixing, compared to a typical minimal enrichment gestation system (CONTROL), and to understand the effect of parity order and backfat on the level of the aggression. The overall average number of fights and the initiated aggressive behavior in the three observation days were greater in the CONTROL pens, although within time, we found a significant effect of treatment only on the number of fights performed 3 weeks post-mixing. The effect of backfat thickness revealed an effect only on the observations of initiated aggressive behavior, which was most frequently performed by sow with a low backfat thickness. Parity order did not have a significant effect on the display of any of the aggressive behaviors. These results suggest that aggression at mixing is unavoidable and, indeed, is essential to ensuring the establishment of the dominance hierarchy and thereby achieving group stability. Nevertheless, optimal enrichment materials could have a beneficial effect on at least reducing its frequency.

**Abstract:**

For sows introduced into new groups, the aggressive behavior associated with establishing a social hierarchy represents a period of severe stress. The aim of this study was to investigate the effect of providing sows with an improved pen environment (straw in racks and ropes) on aggressive behavior after mixing and to understand the role played by sow back fat thickness and parity order. At 29 d post-service, sows were mixed into IMPROVED or CONTROL pens with individual feeding stalls (6 groups/treatment, 20 sows/group). Aggressive behavior was recorded for 2 h at mixing (T0) and 24 h (T1) and 3 weeks post-mixing (T21). Overall, the sows in the CONTROL pens performed more fighting behavior compared to the IMPROVED sows (*p* < 0.001). This difference was significant only at T21 (*p* < 0.001). Additionally, the sows in the CONTROL pens generally initiated more aggressive behaviors than the sows in the IMPROVED pens (*p* = 0.02). The sows with a low back fat thickness initiated more aggressive behaviors, but parity had no significant effect on any of the aggressive behaviors. These results indicate a beneficial effect of improvements to the pen environment on the aggression performed by group-housed sows between the time of mixing and three weeks later. The effect was reduced on the day of mixing, which is in accordance with the necessity for sows to employ aggression to establish the dominance hierarchy.

## 1. Introduction

In current indoor gestation housing systems, sows experience several highly relevant stressors with welfare consequences. The major stressor for pregnant sows is being mixed into new groups [1,2]. The associated aggression required for sows to establish the social hierarchy often has detrimental implications for sow welfare and production. In light of this and of the increased use of group housing systems for sows in response to the forthcoming European ban on stalls, as called for by the European Citizens’ Initiative “End the cage age” [3], finding methods that reduce this stress would be necessary nowadays.

Most agonistic behaviors resulting from mixing unacquainted groups of pigs occur within the first hours after mixing, and the dominance order tends to be stable within 48 h [4,5]. Agonistic behavior includes offensive and defensive contact and non-contact events, such as fighting, biting, pushing, and pursuing, as well as communicative elements such as threats expressed through body movements and vocalizations [6]. There are various measures that are effective in mitigating this aggression, such as the use of a specialized mixing pen, increasing the space allowance, or the provision of enrichment materials [7]. Environmental enrichment effectively reduced post-mixing aggression in weaner and finisher pigs [8,9,10,11]. However, the impact of environmental enrichment on sow aggression is less investigated [12,13], especially around mixing, and the limited literature works that are available report contradictory findings. Previous studies found that the provision of straw in individual racks for sows housed in small static groups with individual feeding cubicles [14] or of point-source materials in floor-fed pens of 12 sows [15] had no effect on aggression at mixing. Meanwhile, Durrell et al. [16] reported that sows housed in small static groups with individual feeding stalls with spent mushroom compost in suspended racks showed a lower frequency of overall agonistic behavior on the day of mixing than sows in barren pens. In contrast, Stewart et al. [17] found higher levels of post-mixing aggressive behavior when sows in a large dynamic group had access to straw provided in a single rack.

Indeed, Commission Recommendation (EU) 2016/336 [18] recommends that all pigs in a group should be able to access materials that are edible or feed-like, chewable, investigable, and manipulable. Straw is a valued material, as it has all the necessary characteristics to be effective and should satisfy the need to express foraging motivation [19,20,21]. However, if it is only provided in limited amounts/circumstances to a large group of sows, it could be perceived as a limited resource and increase aggression between sows [17]. In the present study, straw was provided at three different locations in the loose area of a pen where 20 sows each had access to their own feeding stall. In addition, we suspended a length of natural fiber rope in each stall such that all sows in the group had individual access to a pliable and easily destructible material.

Animal factors such as body weight/size and age (e.g., sow parity) influence a pig’s position in the dominance hierarchy. Some studies found a positive correlation between social rank and weight and parity [22,23,24,25], while others found no correlation [4] or a negative correlation [6]. However, only a few studies [26,27,28,29] investigated the impact of these parameters on aggressive behavior, and these report contradictory results. Considering that the individual level of aggressiveness is not necessarily correlated with the pig’s dominance rank [4,30], there is a need to better determine the role of animal factors in sow aggressive behavior.

We hypothesized that sows housed in pens and provided with straw in racks and manila ropes, in addition to two blocks of wood and two chains, would engage with these materials, which would function as distractions to reduce aggressive behavior during hierarchy establishment. To test the above hypothesis, aggressive interactions were recorded immediately after mixing and 24 h and 3 weeks post-mixing. Moreover, we also investigated the role played by back fat thickness and parity order on the level of aggressive behavior and potential interactions with environmental complexity.

## 2. Materials and Methods

This study was conducted with the approval of the Teagasc Animal Ethics Committee (Approval no. TAEC 2020-266). It did not require licensing under the European Communities Regulations (2002), as no invasive measures were used.

### 2.1. Animals and Treatments

The study was carried out on a commercial 2000-sow farrow-to-finish farm in Co. Cork, Ireland, as part of a larger study described by Lagoda et al. (under review). The study was performed in six replicates using batches of sows weaned between July 2021 and November 2021. In total, 240 Large White X Landrace sows were used in the study. The sows were artificially inseminated in stalls in the service house within 24 h of displaying signs of oestrus post-weaning and remained in the stalls without environmental enrichment until day 28 post-insemination. On day 25 post-insemination, 40 sows within the replicate were selected for the experiment in such a way that both treatments were balanced according to the parity order (parity 1–5, mean ± standard deviation; 2.4 ± 1.03) and back fat thickness. The experiment started on the day that the sows were moved to gestation pens and mixed (day 28.9 ± 0.37 post-insemination) into one of the two following experimental treatments:

CONTROL: A total of 20 sows per replicate were moved to a fully slatted gestation pen with two rows of ten individual free-access feeding stalls, in which two blocks of wood on chains and two simple chains were provided as enrichment in the middle of the loose slatted area. IMPROVED: A total of 20 sows per replicate were moved to similar pens but in which the floors of the feeding stalls were covered with rubber mats (EasyFix Rubber Products, Ballinasloe, Galway, Ireland). In the middle of the loose area, there were two blocks of wood on chains and two simple chains, and a portion of the floor was covered with rubber mats, in the middle of which a rooting tower holding straw was mounted. A straw rack was also mounted on the gates of the pen at either end of the loose area and suspended above a steel collection plate on the floor. Manila ropes (1 m manila rope; Marine Suppliers & Co., Ltd., Howth, Dublin, Ireland) were suspended at a height of one meter within each feeding stall. The racks were filled with straw each day throughout the trial, while manila ropes were replaced as often as necessary to provide continuous access.

In each pen, the space allowance per sow was 2.62 m²/sow, and 20 individual free-access feeding/lying stalls (0.55 m width × 2.3 m length) were available. The sows were free to move around the remainder of the pen (7.2 m width × 7.3 m length; loose roaming area between two rows of feeding stalls: 2.7 m width × 7.3 m length), and they had free access in and out of the feeding stalls through a rear closing gate which could also be locked into position (open or closed) using a valve lever.

The sows were fed a standard, restricted gestation diet twice daily (Table 1), while drinking water was available ad libitum with a ratio of 3 drinkers/20 sows per pen.

All sows were maintained in their treatment group until approximately 110 d of gestation, when they were moved into the farrowing crates.

### 2.2. Aggressive Behaviors

Aggressive behaviors were monitored by two trained observers, who alternated between the two treatments in each replication, at three times: immediately after mixing (T0), 24 h post-mixing (T1) and 3 weeks post-mixing (T21). All sows were identified by individual marks on their backs. Before the observations commenced, the animals were allowed 10 min to get used to the presence of the observer, who was positioned in the corridor outside the pen. During the observation days, all occurrences of various aggressive behaviors and the identities of the sows involved in the encounters were recorded for 2 h continuously from 8:00 to 10:00. The list of aggressive behaviors recorded was adapted from Stewart et al. (2008) [17] and is summarized in Table 2. For each behavior, except for fighting, the sows receiving the aggressive behavior were also identified. The aggressive behaviors of biting, head knock, and chase were merged for the analysis and defined as “initiated” behaviors, while bitten, head knocked, and chased were considered as “received” behaviors.

### 2.3. Back Fat Thickness

The back fat thickness (BFT) was recorded at 25 days post-service. BFT was measured at the last rib, 6 to 7 cm off the midline on the left and right side (P2 position), using an ultrasonic scanner (Lean-meter, Renco, Minneapolis, MN, USA).

### 2.4. Statistical Analysis

All the behaviors recorded (fight, initiated and received aggressive behavior, threat, and avoidance) were calculated as the number of events per hour. For the purposes of the analysis, the sows were categorized on the basis of the BFT of the sows within each pen in “LOW”, “MEDIUM”, and “HIGH” sows. “MEDIUM” sows were the animals that belonged to the interquartile range of their pen (8.5–14.5 mm), while “LOW” and “HIGH” sows were the animals that belonged to the lower (5.5–10 mm) and upper (12.5–21 mm) quartiles of their pen, respectively. Regarding parity order, the sows were divided into young (parity 1–2) and old (parity 3–5).

The statistical analyses were carried out using the software package SAS (SAS Institute, Inc., Cary, NC, USA). Normality tests of data distribution and residuals were performed for every variable evaluated with the PROC UNIVARIATE using the Shapiro–Wilk test. None of the variables recorded in the study were normally distributed; therefore, the GENMOD procedure with Poissson distribution was used for the data processing. The model considered the pen as the experimental unit, while the replicate, day of measure, treatment (control vs. improved), back fat thickness (low vs. medium vs. high), and parity order (young vs. old) and their interaction were considered as fixed effects. The pen by day was considered as a repeated effect. For all statistical tests, the significance level was established at *p* < 0.05.

## 3. Results

**Figure 1:**
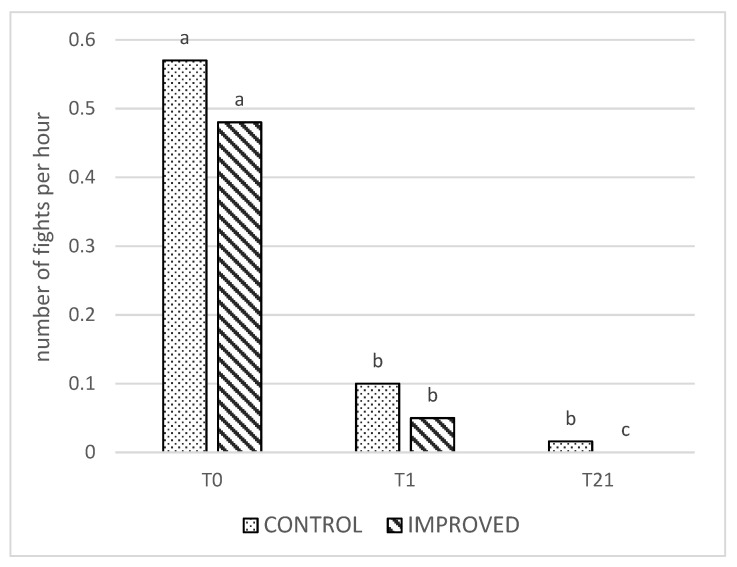
Number of fights per hour in the CONTROL and IMPROVED pens on the day of mixing (T0), the day after mixing (T1), and three weeks later (T21). a, b, c: Different letters indicate significant differences (*p* < 0.05) between the treatment and time.

Overall, there was an effect of treatment on the average number of fights, with the sows in the control pens performing more fighting behavior compared to the improved sows (*p* < 0.001). Within time, this difference was significant only at T21 (*p* < 0.001, Figure 1). Moreover, there was an overall effect of time, with more fights on T0 compared to T1 and T21 (*p* < 0.001). However, in the control pens, a similar number of fights were recorded on T1 and T21, whereas the number of fights decreased significantly between T1 and T21 in the improved pens (*p* < 0.001; Figure 1).

**Figure 2:**
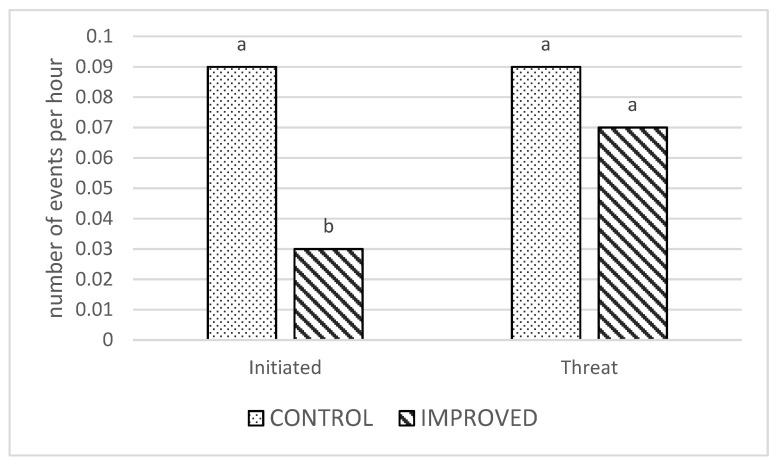
Number of initiated aggressive behaviors and threats per hour in the CONTROL and IMPROVED pens. a, b: Different letters indicates significant differences (*p* < 0.05) between treatments.

There was an effect of treatment on the average number of initiated aggressive behaviors, being higher in the control pens compared to the improved pens (*p* = 0.02; Figure 2). There was no treatment effect on threats and no interactive effects for either variable (*p* > 0.05).

**Figure 3:**
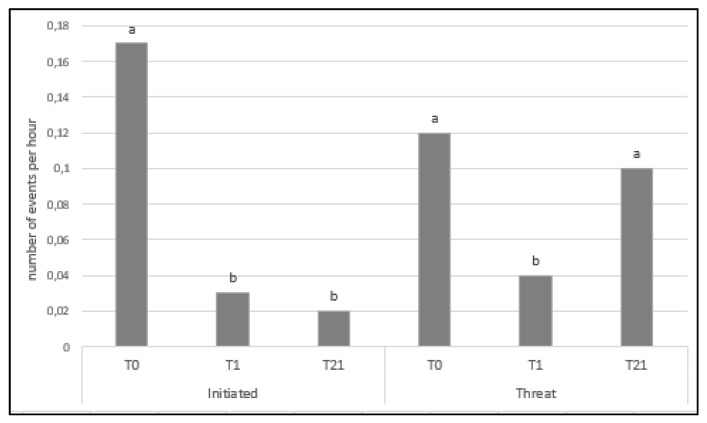
Average number of initiated aggressive behaviors and threats per hour according to the time after mixing: the day of mixing (T0), the day after mixing (T1), and three weeks later (T21). a, b: Different letters indicate significant differences (*p* < 0.05) over time.

Moreover, there was an effect of time on both initiated aggressive and threat behaviors. There were more initiated aggressive behaviors on T0 than on any other day (*p* < 0.001; Figure 3). There were more threat behaviors on d0 and d21 (*p* = 0.002; Figure 3).

There was no interaction between treatment and back fat thickness regarding the number of any of the different aggressive behaviors (*p* > 0.05). However, regardless of the environmental complexity, there was an effect of back fat thickness on the expression of initiated aggressive behaviors (Table 3), which were performed more by low-BFT sows (*p* < 0.0001).

No interaction was found between treatment and parity order. Regardless of the environmental complexity, the parity order did not have a significant effect on the display of any of the aggressive behaviors (*p* > 0.05).

## 4. Discussion

The present data support our hypothesis that improvements to the environment of a group housing system with free-access stalls reduces aggressive behavior including fighting, likely mediated by the distraction provided to the sows by good environmental enrichment. Given the need for sows to employ aggression to establish the dominance hierarchy, the reduced effect on aggression on the day of mixing is perhaps unsurprising.

Regarding fighting behavior, the comparison between treatments revealed significant differences overall and specifically on d21, confirming the hypothesis that the sows in the improved pens would fight less frequently than the sows in the control pens. However, on d0 and on d1, despite the sows performing numerically fewer fights, the difference was not significant. This is likely because the dominance hierarchy was still in formation and is in line with previous research with sows [14,15,16]. These studies and the current study confirm that, irrespective of the housing system, fighting at mixing is mostly unavoidable, and it is an essential component of the establishment of the dominance hierarchy [14]. Indeed, given the evolutionary importance of the dominance hierarchy to group stability [4], it is not surprising that it takes precedence over other less valuable activities, such as, in this case, interacting with the enrichment materials or even eating the straw. This is also in spite of the sows used in the study not having prior experience with such materials, which would likely have heightened their interest in them [31]. Our findings suggest that it is not possible to influence sows fighting at mixing by providing these materials as a distraction [15].

However, it is important to note that, 21 days after mixing, there were significantly more fights in the control pens than there were in the improved pens, and the reduction in the frequency of fights between day 1 and day 21 was faster in the latter pens. This indicates that environmental enrichment has an important role later, when the social hierarchy was established. This is in contrast to Durrell et al. [16] and Greenwood et al. [15], who found no differences between barren and enriched pens in terms of the number of fights observed between day 2 and day 14/20 after mixing. The type and the number of materials provided may be responsible. In the study of Greenwood et al. [15], no foraging substrate, such as straw, was provided, and the number of ropes was lower than the number of sows in the pen, probably decreasing the attractiveness of this enrichment material. Additionally, Durrell et al. [16] only used one form of enrichment, whereas both straw and natural fiber ropes were used in the current study.

As already shown by previous studies [15,16], it is clear that, regardless of the material provided, fights are frequent on the day of mixing and decrease considerably thereafter. This result is consistent with other findings, namely, the finding that, in domestic sows, the dominance order tends to stabilize within 24–48 h of mixing [4,26].

Within time, no significant differences were found between treatments in terms of initiated aggressive behaviors. However, the overall number of these behaviors was significantly lower for sows in improved pens. In contrast, Greenwood et al. [15] found that the material provided (ropes, plastic disk swings, and rubber mats) had no effect on bites and head knocks performed by sows. Durrell et al. [16] found a significant difference on day 1 after mixing in the frequency of agonistic behavior, particularly the behavior “head-thrusting”, with the sows in the barren pens showing a higher frequency than the sows in the enriched pens. Although fighting and aggressive behaviors were not distinguished separately by Stewart et al. [14,17], they showed that the provision of straw to sows in large dynamic groups increased the average proportion of aggressive behavior, while access to straw had no effect on the occurrence of aggressive behavior in the post-mixing period in small static groups. Regardless of the experimental treatments, initiated aggressive and threat behaviors showed a different trend over time. Initiated aggressive behavior decreased soon after the day of mixing, while threat behaviors showed the same frequency on d1 and on d21. These findings suggest that once the social hierarchy is established, sows replace aggressive behavior with threat displays.

Regardless of the environmental complexity, there was an effect of back fat thickness on the expression of aggressive behavior only for initiated aggressive behaviors, which were performed more by low-BFT sows. This finding is in contrast with the few studies that investigated the role of body condition in determining the outcomes of aggression in pigs. Indeed, Andersen et al. [27] and D’Eath et al. [28] reported that heavier pigs initiated more aggressive acts and were more involved in fighting. Conversely Mount et al. [26] found no correlation between the number of aggressive interactions initiated and received and body weight. So far, the scientific literature has mainly focused on the correlation between weight and the position of animals within the dominance hierarchy based on the success in winning agonistic interactions [6,22,23,24,25]. The limited number of studies that have investigated the correlation between body condition and frequency of aggressive behavior makes it difficult to find an explanation as to why low-BFT sows initiated more aggressive behavior. However, a possible explanation could lie in the fact that fatter sows may engage in less aggressive behavior because their size alone scares the other sows away.

Regardless of the environmental complexity, the parity order did not have a significant effect on the display of any of the aggressive behaviors. This is in line with the findings of Mount et al. [26]. By contrast, Strawford et al. [29] found that old sows were involved in a greater number of aggressive encounters than young and intermediate sows. In light of the small differences found in the present study in terms of the frequency of aggressive behavior depending on back fat thickness and parity order, it seems that, in pens with free-access, full-length individual feeding/lying stalls, these intrinsic factors have a marginal impact on aggressive behavior.

## 5. Conclusions

This study confirms the benefit to sow welfare, through reduced aggression, of improving the housing environment. The benefit was likely largely driven by the sows’ interest in the substrates provided. The findings indicate that aggression at mixing is unavoidable, as it is essential for the establishment of the dominance hierarchy and group stability, but enrichment materials could at least have an effect on reducing its frequency. The current study indicates that the body condition (measured as back fat thickness) and parity do not have a great impact on sow social behavior, even though the thinnest sows were those that most frequently performed initiated aggressive behaviors.

## Figures and Tables

**Table 1:** Ingredients and composition of the gestation diet.

INGREDIENT	%
Barley	41.1
Maize	7
Wheat	23
Suguar beet pulp	4
Hi pro soya	15
Soya hulls	4
Soya oil	2.5
Min	2.4
**COMPOSITION**	
C Protein	15.6
C Oil	4.9
C Fiber	5.3
C Ash	5.7
De	13.3
Lysine	0.9
M + C	0.53
Threonine	0.61
Calcium	0.95
Av Phos	0.36
Salt	0.58

**Table 2:** Description of aggressive behaviors recorded in the study (adapted from Stewart et al., 2008 [17]).

Behavior		Description
Fighting		Mutual pushing parallel or perpendicular; ramming or pushing of the opponent with the head; with or without biting in rapid succession. Lifting the opponent by pushing the snout under its body
Initiated	Biting	Biting any part of another sow, but not as part of a head knock
	Head knock	Ramming or pushing another sow with the head (with or without biting)
	Chase	Moving rapidly in pursuit of another sow
Received	Bitten	Being bitten by another sow, but not as part of a head knock
	Head knocked	Being rammed or pushed by another sow with the head (with or without biting)
	Chased	Moving rapidly/running away from another sow
Threat		Interaction expressed through body movements without physical contact, with a sow actively withdrawing (avoid)
Avoid		Active withdrawal of a sow being threated without physical contact

**Table 3:** Number of aggressive behaviors per hour according to back fat thickness (low, medium, and high).

Behavior	Back Fat Thickness			*p*
	Low	Medium	High	
Fight	0.003 ± 0.001	0.002 ± 0.000	0.002 ± 0.000	NS
Initiated	0.13 ^a^ ± 0.03	0.06 ^b^ ± 0.01	0.02 ^b^ ± 0.01	<0.001
Received	0.06 ± 0.02	0.06 ± 0.01	0.05 ± 0.02	NS
Threat	0.10 ± 0.02	0.05 ± 0.02	0.09 ± 0.02	NS
Avoid	0.05 ± 0.02	0.09 ± 0.01	0.06 ± 0.02	NS

^a, b^: Different letters within a row indicate significant differences between means.

## Data Availability

The data presented in this study are available on request from the corresponding author.
